# Maximizing the impact of megaprojects: Urgent implications of financial inclusion drive for effective anti-poverty measures

**DOI:** 10.1016/j.heliyon.2024.e39658

**Published:** 2024-10-22

**Authors:** Salman Mahmood, Liangang Zhang, Shoaib Aslam, Navid Khan

**Affiliations:** a Faculty of Economics and Management, Southwest Forestry University, Kunming, Yunnan, China; b Department of Commerce, The Islamia University of Bahawalpur, Pakistan; c Faculty of Management and Economics, Kunming University of Science and Technology, Kunming, Yunnan, China

**Keywords:** CPEC, Poverty reduction, Inclusive growth theory, Social exchange theory, And financial inclusion

## Abstract

Megaprojects are becoming more and more essential for poverty reduction and inclusive development. Regrettably, the CPEC mega project has yet to yield successful outcomes in Pakistan. The incorporation of financial inclusion (FI) has the potential to catalyze its outcomes. Despite abundant studies on advanced CPEC applications and poverty reduction techniques, the relationship between FI and CPEC has been the subject of relatively little research. Therefore, we analyzed CPECD and FI, specifically emphasizing economic opportunity, growth, and poverty reduction (PR). The study accounted for the viewpoints of small and medium-sized enterprises (SMEs). Using Amos 24 and the Process Macro, we analyze the mediational and moderated mediational models. The findings show that CPECD reduces poverty. Opportunity and growth both significantly influence the outcome. FI practices enhance the opportunity and growth link between CPECD and PR. Therefore, it is imperative to address the issue of FI shrinkage, as it could impede the efforts of SMEs to promote inclusive growth and alleviate poverty.

## 1 Introduction

Pakistan has been aspiring for high and sustainable economic growth for decades. Although it has made tremendous progress in restoring macroeconomic stability, much work remains to boost socio-economic growth [1]. Policymakers are developing many development ideas in this regard. One of them is the China-Pakistan Economic Corridor (CPEC), an extension of the One Belt, One Road (OBOR) initiatives [2]. It is a multi-billion-dollar project that aims to connect the two countries through a network of highways, railways, and pipelines. This route connects Pakistan to China, Afghanistan, and Central Asia [3,4]. China launched CPEC in 2013 to improve Pakistan's economic infrastructure [5]. It was intended to boost regional cooperation and trade between South Asian countries and was aptly described as a game-changer for Pakistan. SMEs and large enterprises expected CPEC to explore new markets for their products, leading to improved economic growth and poverty alleviation [6].

Although CPEC offers attractive prospects for foreign businesses, it has become a significant challenge for SMEs in Pakistan to secure financing to avail themselves of these opportunities [7]. The financial inclusion drive can enhance the availability of these options [8]. The term "financial inclusion" was defined by Sarma and Pias [9] as ensuring that all members of an economy can easily access, use, and benefit from the formal financial system. Accessibility, availability, cost, and utilization are only a few of its multiple components [8]. The plan for CPEC was to create a business ecosystem and draw in local and foreign enterprises [7]. However, Pakistan's poor financial inclusion impedes attracting domestic investment, contributing to low economic growth [10]. Creating a business ecosystem and reaping the benefits of CPEC requires more than ignoring financial inclusion and relying solely on Chinese loans and investments [11]. Consequently, the ideal level of CPEC is still being explored.

In this area, Pakistan's fragile economic situation also calls for attention. Addressing the numerous challenges, including weak infrastructure, security issues, energy crises, and poverty, through CPEC [12] will assist in moving toward Sustainable Development Goals (SDGs) [[13], [14], [15]]. However, until CPEC optimizes through financial inclusion, 8 out of 17 SDGs remain unattainable. As a result, the current study, using inclusive growth theory, evaluates SMEs' perceptions of the interaction of CPEC developments with financial inclusion and poverty alleviation in Pakistan.

**Fig. 1:**
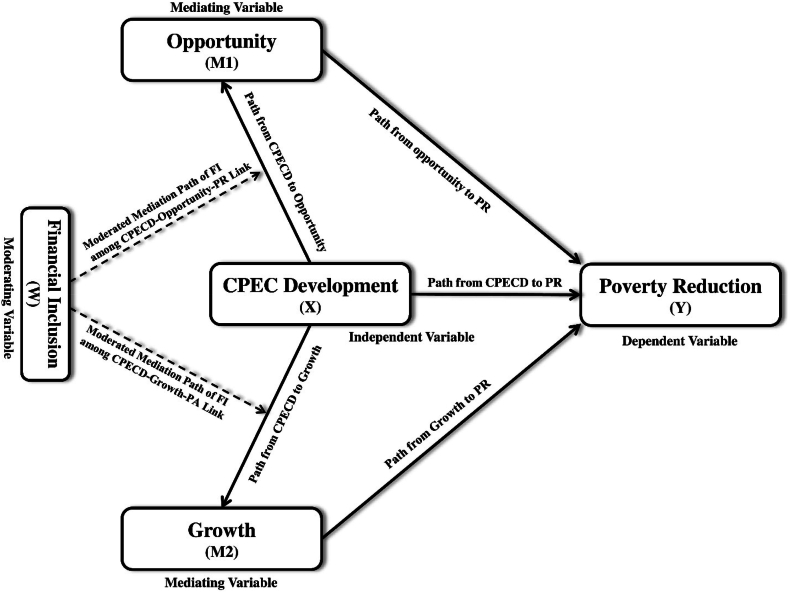
Relationships between Variables Note: CPECD = CPEC development FI = financial inclusion, and PR = poverty reduction.

Several studies have evaluated CPEC's influence on economic development, employment, and livelihood [7,[16], [17], [18], [19]]. Specifically, Raza et al. [20] mentioned the economic gains to Pakistan from the CPEC initiatives. Kanwal et al. [21] investigated the local community's support for the CPEC's development and found a link between community support and benefits. Ali et al. [22] emphasized how CPEC improves the local community's education, income, and job prospects. Anwar et al. [23] underlined the association between economic zones and industrialization. However, they ignore the role of local communities in various opportunities, growth, and poverty reduction. Security challenges, the development of Gwadar port, energy crises, and Pakistan's business and employment prospects have all been the subject of previous theoretical work relating to CPEC [20,23,24]. The administration's or stakeholders' perceptions of CPEC's benefits to local populations dominate previous literature, leaving financial inclusion out of the discussion. However, financial inclusion is a tried-and-true approach to alleviating poverty and attaining socio-economic stability. A more detailed examination of the mechanisms that could determine the success or failure of these megaprojects is required. Furthermore, we have yet to investigate the constraints that impede megaproject advantages and promote elite capture, particularly in emerging economies. Additionally, the mediating variables of opportunity and growth and the moderating variable of financial inclusion, as viewed through the lens of inclusive growth theory, have escaped. As a result, the researcher has identified a conceptual research gap. Consequently, this study aims to answer the following research question: *Do CPEC development and financial inclusion trigger opportunity, growth, and poverty reduction?* We developed five objectives to address the research question. (I) to evaluate the effect of CPECD on poverty alleviation in Pakistan, and (II) to determine opportunity's mediation role. (III) to assess growth's mediation role. The objective of (IV) is to ascertain the moderated mediational impact of financial inclusion on the relationship between CPECD, opportunity, and PR in Pakistan. Finally, (V) aims to assess the moderated mediational effect of financial inclusion on the relationship between CPECD, growth, and PR in Pakistan. To attain these research objectives, we employed structural equation modeling (SEM) and ordinary least squares regression (OLS) to account for mediating and moderating effects. Fig. 1 illustrates the interdependencies and predictability of the numerous constructs.

**Fig. 2:**
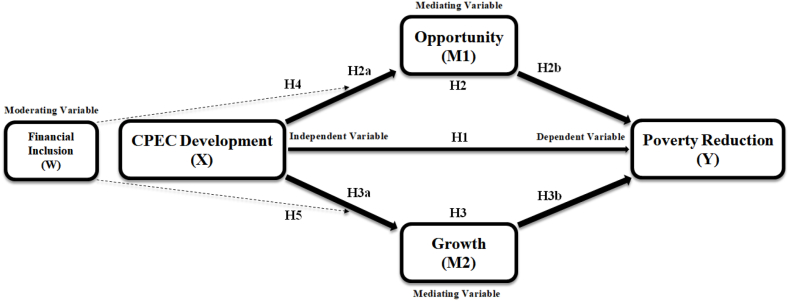
Theoretical Model Note: H6 will calculate the total effects of the entire mediational model.

Fig. 2 provides additional information concerning the proposed relationships.

## 2 Literature review and hypotheses development

### 2.1 Theoretical framework

Theoretically, this study adds to social exchange and inclusive growth theories. The premise of social exchange theory (SET) is that people form and maintain connections because they hope to benefit from them [25]. According to SET, if local populations believe a development project would benefit them in terms of societal progress, they will support it and endorses its development for its advantages [26]. To gain acceptance and ownership, the government must educate local people about the pros and cons of CPEC projects and include local stakeholders in the decision-making process [5]. The justification for using this theory stems from the opportunities, growth, and reciprocal benefits of CPEC for both nations. The CPEC offers a thriving economic climate, generates both local and international investment, and alleviates poverty. On the contrary, inclusive growth theory aims at alleviating poverty by promoting an equitable distribution of the advantages of economic growth. The approach seeks to create a more balanced and sustainable economic system that benefits everyone, particularly those most vulnerable to poverty and inequality. In particular, inclusive growth reduces the tradeoffs between economic progress and inequality by empowering the impoverished as consumers, employees, owners, suppliers, and community members. Our study fundamentally bases this theory on the essence of megaprojects. Multilateral projects such as CPEC, in conjunction with financial inclusion, guarantee equitable allocation of advantages to all disadvantaged groups and sustained alleviation of poverty. The Asian Development Bank [27] specifically formulated this theory in 2007 to address the issue of "growth without development." The term "growth without development" refers to a phenomenon that arose from classical growth theory developed by British economists during the Industrial Revolution [28]. Despite making economic progress towards the end of the 20th century, developing nations still need to achieve the anticipated reduction in poverty. Instead, this growth has resulted in income inequality, resource depletion, environmental degradation, and social conflicts. Furthermore, this theory prioritizes increasing the output of goods and services to achieve economic growth without considering the broader consequences for social and economic progress. In simpler terms, without gains in healthcare, education, and social welfare, economic growth constitutes growth without development [29].

Public and corporate policy recognizes the importance of inclusive growth. Following this, China has been promoting inclusive growth for a significant period. According to Wang et al. [30], inclusive growth from the Chinese perspective is a novel economic development concept that prioritizes the principles of coordination and sharing so that all individuals can contribute to and benefit from economic growth. According to Nari Kahle et al. [31], the Indian government is committed to attaining "inclusive growth" through its economic policies and strategies. Focusing on inclusive growth reflects the government's recognition of reducing inequality and fostering social cohesion for sustainable development. In line with this, Mahmood et al. [8] conducted an inclusive growth-based study in Pakistan. This study's findings show how Pakistan can achieve inclusive growth through FI. They demonstrated that providing access to credit is a direct solution to inclusive growth.

Financial inclusion initiatives under CPEC projects may become critical for equitable and inclusive growth. It can make people competitive in terms of economic opportunities. Conversely, financial exclusion reduces people's ability to achieve the minimum living standards, pushing them to the margins of society [32,33]. Hence, based on the above arguments, boosting inclusive growth requires people to access safe and inexpensive financial services through either "supply-leading" or "demand-following" channels.

### 2.2 CPEC development and poverty reduction

The establishment of the CPEC aimed to cultivate a conducive business climate that would successfully allure both domestic and international investments [7,34,35]. The smooth and uninterrupted influx of investments into Pakistan has the potential to foster growth in various socioeconomic areas, including industrial, education, healthcare, and social welfare. Over the years, studies on CPECD have represented localized communities and large cooperations. However, a limited body of work focuses on experiences and challenges in how SMEs improve productivity and competitiveness through CPEC. For example, Xie et al. [36] have suggested that the CPEC has the potential to impact Pakistan's development and industrialization positively. As Wasim & Siddiqi [37] highlighted, the CPEC has increased industrial productivity in Pakistan. Specifically, the cement and steel industries have experienced notable growth. As a result, this helped to create new businesses, jobs, and a higher standard of living for Pakistanis, contributing to the country's overall development. Thus, the CPEC project could boost Pakistan's economy and reduce poverty [38]. It can significantly enhance business growth, particularly for small enterprises [39]. Based on the preceding debate, the current analysis reveals that CPEC development considerably affects residents' poverty.

### 2.3 The mediating role of opportunity

The concept of opportunity pertains to the possible advantages an individual or group can leverage to achieve economic success [8]. The entrepreneur can identify and capitalize on market opportunities that have the potential to give rise to new business ventures [40]. According to Johansen et al. [41], financial institutions are vital to a nation's economic development. They are widely acknowledged as key players in providing economic opportunity to individuals and society. Also, the scale of the investments involved in the CPEC projects is significant, which underscores the importance of these initiatives in generating millions of job opportunities and addressing poverty in the region [21,42,43]. In other words, the CPEC comprises multiple economic zones to promote corporate growth and further Pakistan's economic development. Hadi et al. [42] support this view that economic corridors strategically promote and enhance entrepreneurial activities and economic growth. Therefore, adding financial support to CPEC investments can provide the desired outcomes. This outlook highlights the obstacles SMEs may encounter in obtaining opportunities without megaprojects and addresses the gap in the literature as viewed through the lens of inclusive growth theory.

### 2.4 The mediating role of growth

Growth is the process of increasing the size of the economy by ensuring that all individuals have equal conditions [8,15]. The CPEC project is believed to positively impact the growth and development of the local population and economy. However, the local population's opinion of CPEC is still being determined, whether the local population sees CPEC favorably or negatively. For instance, although some locals are optimistic that the project will end poverty and raise their standard of living [22], others are pessimistic and fear it will hurt their local businesses [18]. The statement implies that the perspectives discussed adhere to social exchange theory's tenets.

China, Pakistan, and the region were anticipated to benefit significantly from CPEC's development. The initiative was expected to create hundreds of thousands of jobs, various markets for commodities and services, and logistics and supply chains for valuable products, all of which would be available to the community with minimal effort and expense [10,44]. Local farmers and SMEs like workshops, hotels, restaurants, supermarkets, gas pumps, and parking facilities were expected to benefit residents [45]. This perspective aligns with the principles of inclusive growth theory—the idea that economic growth should be inclusive and benefit all members of society.

Indeed, the current mega-economic activity represents Pakistan's best chance to break the country's cycle of low growth and improve its citizens' living standards by reducing poverty levels [46,47]. A condition in which an individual or group lacks the financial resources and necessities for a basic level of existence [48]. Upon careful consideration of various arguments, we assume that the development of CPEC significantly impacts growth and possesses anti-poverty capabilities. However, SMEs' role in various growth and poverty reduction activities is ignored. As a result, an empirical examination of these topics is critical since it provides policy adjustments to combat poverty and achieve socio-economic sustainability.

### 2.5 The moderating role of financial inclusion

The CPEC is a continuation of the twenty-first-century maritime Silk Road. Experts agree that China's massive investment ambitions in Pakistan are a "game-changer" in regional and global economic development. Empirical research on CPEC, however, demonstrates that local community investment is rare [10]. Financial constraints are at the root of this issue; therefore, offering access to diverse financial services through FI can offer a better solution [38]. Basic accounts, interest-free loans, soft loans, microcredit, export credit, and deposit facilities are among the financial goods and services that address customers' varied financial demands [2,8]. The projects under the China-Pakistan Economic Corridor (CPEC) and the financial inclusion push offer potential for local residents to engage in entrepreneurship, contributing to the reduction of poverty. However, Pakistan faces challenges in effectively managing its public finances, which is the main factor contributing to economic shocks such as unemployment and poverty in the local population. Regrettably, this is the reason why CPEC has not yet achieved its maximum capacity [10]. Consequently, the majority of Pakistanis, particularly the local population, consider it a failure [18,43]. They believe that the CPEC still needs to deliver on its promises.

Well-structured financial institutions are essential for taking advantage of CPEC's development and associated opportunities [10,49]. Identifying lasting economic growth under CPEC requires addressing potential obstacles, specifically the level of financial inclusion [10,38]. The well-documented literature advocates the utility of FI and stresses that it may assist in achieving optimal CPEC levels by establishing new business ventures and creating jobs. In summary, if this trade route operates with increased financial inclusion through local incentives, it will present a significant opportunity for the indigenous population in terms of diverse livelihoods and local economic prosperity. Otherwise, the local community will miss out on the current opportunities [10]. In these critical circumstances, financial inclusion might benefit the CPEC's success, job creation, and long-term, inclusive economic growth in Pakistan.

We mapped out a conceptual framework (Fig. 2) to establish causality and see how explanatory variable CPEC development affects explained variable poverty reduction. The framework identifies critical variables such as opportunity and growth as mediators and financial inclusion as the moderator between CPEC development and its impact on poverty reduction.

Based on the conceptual framework, this research outlines the following hypothesis.

H1 CPEC development significantly impacts poverty reduction in Pakistan.

H2 Opportunity mediates the relationship between CPEC development and poverty reduction, such that:

H2a CPEC development significantly and positively impacts opportunity.

H2b Opportunity significantly and positively impacts poverty reduction.

H3 Growth mediates the relationship between CPEC development and poverty reduction, such that:

H3a CPEC development significantly and positively impacts growth.

H3b Growth significantly and positively impacts poverty reduction.

H4 Financial inclusion moderates the mediated relationship of opportunity between CPEC development and poverty reduction.

H5 Financial inclusion moderates the mediated relationship of growth between CPEC development and poverty reduction.

Also, if the direct link between CPECD and PR remains significant after adding the mediators opportunity and growth, it indicates partial mediation. Nevertheless, if the direct relationship between CPECD and PR becomes insignificant, this indicates full mediation. Hence, we hypothesize that.

H6 There exists partial mediation between CPEC development and poverty reduction.

These hypotheses ensure a systematic approach to the research questions and objectives and closely align with the conceptual model.

## 3 Methodology

### 3.1 Measurements

Like any cross-sectional study, we adapted all scale items from prior research (see Table 1). To measure CPEC development, we borrowed a scale with five items from Ali et al. [50] and Saad et al. [7]. We adapted scales of opportunity and growth, each containing seven items, from Mahmood et al. [8]. Financial inclusion was measured using a 5-item scale developed by Rastogi [51]. Finally, poverty reduction was measured using a 5-item scale developed by Saad et al. [7]. Table 1 displays all the variables and their roles. The Cronbach's alpha for CPEC development, opportunity, growth, financial inclusion, and poverty reduction scales were 0.978, 0.965, 0.947, 0.940, and 0.926, respectively. All the scales used in this study are well-validated and fall within the standard range. Fig. 2 presents the theoretical model for the current study.

**Table 1:** Measurements.

Variables	Symbols	Source	Description
**Independent variable**			
CPEC Development	CPECD	Primary	The five-item CPEC development measurement scale is adapted from Ali et al. [50] and Saad et al. [7].
**Mediating variables**			
Opportunity	OT	Primary	The seven-item opportunity measurement scale is adapted from Mahmood et al. [8].
Growth	GT	Primary	The seven-item growth measurement scale is adapted from Mahmood et al. [8].
**Moderating variable**			
Financial Inclusion	FI	Primary	The five-item financial inclusion measurement scale is adapted from Rastogi [51].
**Dependent variable**			
Poverty Reduction	PR	Primary	The five-item poverty reduction measurement scale is adapted from Saad et al. [7].

### 3.2 Empirical analysis

This study used a cross-sectional design and was quantitative and exploratory. The current study's population consists of SMEs located in eight districts in Pakistan's three regions, including the northern region (Gilgit, Peshawar, and Islamabad districts), the central region (Faisalabad, Sialkot, and Lahore districts), and the southern region (Karachi and Gwadar districts). SME owners are the primary inquiry unit for the current study. There are nearly 5 million SMEs in Pakistan; therefore, to calculate the sample size, we employed the Krejcie and Morgan table [52]. The chambers of commerce provided lists of registered SMEs in the targeted cities, which the Small and Medium Enterprises Development Authority (SMEDA) subsequently verified. We calculated the sample size 384 using Krejcie and Morgan's recommended table. Since we knew the lists of registered SMEs, we used a simple random sampling technique. We also used availability and readiness to respond as the primary criteria.

The data was accumulated over six months, from March 2023 to August 2023. We chose this period for two specific reasons: I). SMEs in Pakistan report their annual financial results after the fiscal year and formulate plans for the following year. This period will enable operational and financial data collection in real-time. II). The China-Pakistan Economic Corridor (CPEC) has reached several important milestones during this period. These milestones have provided valuable opportunities to study the immediate impact on local SMEs and poverty reduction initiatives. A 5-point Likert scale was used in this study (1 = strongly disagree, 5 = strongly agree). We distributed 800 questionnaires and received 432 responses. Due to missing data, 37 were excluded. 395 were utilized in statistical analysis, meeting the standard sample size for structural equation modeling (SEM). 85 % of the 395 participants were male. Also, 115 responses were taken from the northern region, including 26 responses from Gilgit, 47 from Peshawar, and 45 from Islamabad. 167 responses were taken from the central region, including 59 from Faisalabad, 48 from Sialkot, and 60 from Lahore, and 110 responses were taken from the southern region, including 69 from Karachi and 41 from Gwadar.

We conducted a pilot test of the questionnaire with 40 respondents [53] before the main study. After piloting, we eliminated all ambiguous items. We then ascertained the normality of the data using skewness and kurtosis. The data was normal with skewness and kurtosis ranged between −0.168 and 0.071 and −1.376 to −0.949 respectively. Furthermore, we assessed the data's multicollinearity by estimating the variance inflation factor (VIF), and VIF = 5.79 implies no multicollinearity as the threshold value is below 10.0. Lastly, we predicted the hypothesized relationship and tested the presence of common method variance (CMV) using Herman's single-factor test and Pearson correlation. Podsakoff et al. [54] agree that if the researcher collects data from the same respondents for the dependent and independent variables, then CMV is possible. Therefore, we conducted Herman's single-factor test, and the results revealed that the percentage of variance explained by a single factor was 39.320 %, less than 50 % [54]. Hence, there was no CMV in the data. In the second part, the correlation results showed that the relationships between the variables were positive and moderate, ranging from 0.121 to 0.866 and being less than 0.9 (see Table 2) [55], indicating no CMV. We conducted the initial analysis using SPSS 23. We used Amos 24 and Process Macro to analyze the mediational and moderated mediational techniques, respectively.

**Table 2:** Descriptive Statistics and Pearson correlation.

	Mean	S. D	CPECD	OT	GT	FI	PR
CPECD	3.053	1.086	1				
OT	2.958	1.193	0.448∗∗	1			
GT	3.045	0.986	0.866∗∗	0.501∗∗	1		
FI	3.065	1.253	0.121∗	0.125∗	0.149∗∗	1	
PR	3.110	1.005	0.380∗∗	0.446∗∗	0.474∗∗	0.138∗∗	1

∗∗ = p < 0.01, ∗ = p < 0.05, CPECD = China Pakistan Economic Corridor Development, OT = Opportunity, GT = Growth, FI = Financial Inclusion, and PR = Poverty Reduction.

## 4 Results and analysis

We ran the exploratory factor analysis (EFA) on five variables (CPEC development, opportunity, growth, financial inclusion, and poverty reduction). Annexure 1 reports the EFA results.

### 4.1 Structure equation modeling-measurement model

As shown in Fig. 2, the current study provided a moderated mediation model in which financial inclusion moderated the mediation impact of opportunity and growth between CPECD and poverty reduction. To investigate this model, we first examined model fitness, convergent validity, and discriminant validity. We then calculated the mediating effects. Next, we used the PROCESS macro with 5000 bootstrap samples to investigate the moderated mediation model. We finally used Interaction 1.7 to draw the interaction graph.

SEM is better than regression because it can (I) estimate both direct and indirect relationships at the same time, (II) include latent variables, and (III) account for measurement error during the estimation process [56]. Before testing our model, we established the independence of the main model variables with confirmatory factor analysis (CFA) in AMOS 24. We employed a mixture of fit indices—CFI, TLI, and RMSEA—to test the appropriateness of our hypothesized model [57]. RMSEA scores below 0.08 and CFI and TLI values above 0.90 are considered good [58]. Table 3 demonstrates a good fit (X2/df = 2.79, RMSEA = 0.07, IFI = 0.967, CFI = 0.967). The author suppressed all cross loadings less than 0.5 and remaing factor loads greater than 0.50 (ranging between 0.689 and 0.978) and statistically signifcant at p < 0.000. Therefore, we confirmed the independence of the main model variables.

**Table 3:** Confirmatory factor analysis (CFA).

Factor	Standardize loading (*p*-value)	S. E	Critical ratio (t-value)	CR	AVE
**CPECD**	0.936-0.969(∗∗∗)	0.011-0.022	41.582-70.487	0.978	0.898
**OT**	0.858-0.978(∗∗∗)	0.016-0.026	30.683-53.930	0.966	0.852
**GT**	0.846-0.935(∗∗∗)	0.026-0.030	32.105-39.749	0.950	0.826
**FI**	0.851-0.885(∗∗∗)	0.040-0.042	23.128-25.032	0.940	0.757
**PR**	0.689-0.955(∗∗∗)	0.033-0.043	16.915-34.154	0.930	0.728
Here X^2^/df = 2.79, RMSEA = 0.07, IFI = 0.967, CFI = 0.967					

∗∗∗ = p < 0.001, CPECD = IFI = Incremental fit index, CFI = Comparative fit index, CPECD = China Pakistan Economic Corridor Development, OT = Opportunity, GT = Growth, FI = Financial Inclusion, and PR = Poverty Reduction.

Following CFA, we used the Master Validity plugin to verify convergent and discriminant validity [59]. For convergence validity, the composite reliability (CR) and average variance extracted (AVE) must be greater than 0.70 and 0.50, respectively. The CR and AVE values in Table 3 for the study variables (CPECD, OT, GT, FI, and PR) are much higher than their threshold levels, highlighting strong correlations between measures of the same construct. Therefore, convergent validity was demonstrated. Subsequently, discriminant validity was ascertained using two aproaches. First, we establish discriminant validity when the AVE square root surpasses the correlation coefficients [58]. According to Table 4, the square root of AVE is greater than the correlation coefficients, indicating low or insignificant correlations between measures of different constructs. Hence, discriminant validity has been confirmed. Second, the HTMT analysis was conducted. HTMT values below 0.90 indicate that discriminant validity is maintained. Table 5 provides evidence of discriminant validity, as all HTMT values are measured below 0.90.

**Table 4:** Discriminant validity.

Variables	CPECD	OT	GT	FI	PR
CPECD	**0.948**				
OT	0.464^a^	**0.923**			
GT	0.894^a^	0.520^a^	**0.909**		
FI	0.131^c^	0.125^c^	0.147^b^	**0.870**	
PR	0.403^a^	0.474^a^	0.495^a^	0.151^b^	**0.853**

CPECD = China Pakistan Economic Corridor Development, OT = Opportunity, GT = Growth, FI = Financial Inclusion, and PR = Poverty Reduction.

a p < 0.001.

b p < 0.01.

c p < 0.05.

**Table 5:** HTMT analysis.

Variables	CPECD	OT	GT	FI	PR
**CPECD**					
**OT**	0.459				
**GT**	0.851	0.523			
**FI**	0.125	0.131	0.157		
**PR**	0.398	0.47	0.506	0.146	

∗∗∗ = p < 0.001, ∗∗ = p < 0.01, ∗ = p < 0.05, CPECD = China Pakistan Economic Corridor Development, OT = Opportunity, GT = Growth, FI = Financial Inclusion, and PR = Poverty Reduction.

### 4.2 Structure equation modeling-structural model

We used SEM to examine the mediating roles of OT and GT on the influences of CPECD and PR. Before testing hypotheses, model fit showed that our model fits the data well. As shown in Table 6, CPECD had a significant positive effect on PR (β = 0.414, p < 0.001), OT (β = 0.474, p < 0.001), and GT (β = 0.901, p < 0.001). Therefore, we accept H1, H2a, and H3a. OT had a significant positive effect on PR (β = 0.308, p < 0.001), and GT had a significant positive effect on PR (β = 0.556, p < 0.001). Therefore, we accept H2b and H3b (refer to Table 6). Finally, following the Hayes mediational conditions, OT (β = 0.146, p < 0.001) and GT (β = 0.501, p < 0.001) mediate the relationship between CPECD and PR. Therefore, we accept H2 and H3 (refer to Table 7).

**Table 6:** Direct effects.

Hypothesis and Path		Estimate	S. E	C.R	*p*-value	Result
H1	PR ← CPECD	0.414	0.038	9.026	∗∗∗	Accepted
H2a	OT ← CPECD	0.474	0.053	10.689	∗∗∗	Accepted
H2b	PR ← OT	0.308	0.033	6.471	∗∗∗	Accepted
H3a	GT ← CPECD	0.901	0.018	41.337	∗∗∗	Accepted
H3b	PR ← GT	0.556	0.094	5.750	∗∗∗	Accepted
H6	PR ← CPECD	−0.226	0.081	−2.276	0.023	Partial Mediation
Model fit for structural model: X^2^/df = 2.30, RMSEA = 0.06, IFI = 0.992, CFI = 0.992						

∗∗∗ = p < 0.001, CPECD = China Pakistan Economic Corridor Development, OT = Opportunity, GT = Growth, FI = Financial Inclusion, and PR = Poverty Reduction.

OT and GT partially mediate the relationship between CPECD and PR (0.421∗∗∗).

**Table 7:** Indirect effects.

Hypothesis and Indirect Path		Unstandardized Estimate	Lower	Upper	Standardized Estimate	*p*-value	Result
H2	PR ← OT ← CPECD (Indirect Effects)	0.118	0.085	0.156	0.146	0.001	Accepted
H3	PR ← GT ← CPECD (Indirect Effects)	0.407	0.329	0.479	0.501	0.001	Accepted
Model fit for structural model: X^2^/df = 4.212, RMSEA = 0.08, IFI = 0.959, CFI = 0.959							

CPECD = China Pakistan Economic Corridor Development, OT = Opportunity, GT = Growth, and PR = Poverty Reduction.

Adding the mediators OT and GT did not change the significance of the direct relationship between CPECD and PR. However, it became negative (β = −0.226, p < 0.023), which means that there was partial mediation (see Table 6). Therefore, H6 is accepted. Besides, in the presence of mediators OT and GT, the total effect between CPECD and PR is well-regressed (i.e., β = 0.421, p < 0.001, LB = 0.346, UP = 0.488). There is also a significant difference between the direct effect of CPECD on PR (β = 0.414) and the total standardized indirect effect of CPECD on PR (β = 0.647, p < 0.001, LB = 0.567, UP = 0.731). This means that OT and GT are effective mediators.

### 4.3 Moderated mediation model

Because of theoretical considerations and the significant mediation model, we used PROCESS macros Model-7 to study the moderating role of FI through which CPECD affects OT and GT. For Process Macro's moderated mediation test, OT and GT were the outcome variables. According to Table 8, the product of CPECD and FI predicted OT (β = 0.083, p < 0.05) and GT (β = 0.063, p < 0.001). The moderate mediation of OT (with a 2 % regression weight) and GT (with a 2.9 % regression weight) was also significant. Therefore, we accept H4 and H5 (refer to Table 8). Hayes [60] states that a conditional indirect effect exists if the independent variable and the moderator have significant relationships and the bootstrapping confidence intervals do not contain zero.

**Table 8:** Moderated Analyses.

	Estimate	S. E	C.R	*p*-value	Lower	Upper
**Model 1 (OT as DV)**						
Constant	2.010	0.410	4.903	0.000	1.204	2.816
OT ← CPECD	0.238	0.130	1.840	0.067	−0.016	0.493
OT ← Interaction	0.083	0.041	2.046	0.041	0.003	0.163
R-square	0.215	–	–	–	–	–
F-statistics	35.599	–	–	0.063	–	–
ΔR-square(F-statistics)	0.008(4.186)	–	–	0.041	–	–
**Model 2 (GT as DV)**						
Constant	1.113	0.188	5.934	0.000	0.744	1.482
GT ← CPECD	0.595	0.059	10.038	0.000	0.478	0.712
GT ← Interaction	0.063	0.019	3.401	0.001	0.027	0.100
R-square	0.759	–	–	–	–	–
F-statistics	410.475	–	–	0.000	–	–
ΔR-square(F-statistics)	0.007(11.565)	–	–	0.001	–	–
**Model 3 (PR as DV)**						
Constant	1.393	0.147	9.469	0.000	1.104	1.683
PR ← CPECD	−0.128	0.079	−1.622	0.106	−0.284	0.027
PR ← OT	0.236	0.042	5.682	0.000	0.514	0.318
PR ← GT	0.463	0.090	5.144	0.000	0.286	0.640
R-square	0.287	–	–	–	–	–
F-statistics	52.578	–	–	0.000	–	–
**Indirect Effect**						
PR ← OT ← CPECD	0.020	0.010	–	–	0.002	0.041
PR ← GT ← CPECD	0.029	0.011	–	–	0.008	0.053

DV = Dependent Variable, CPECD = China Pakistan Economic Corridor Development, OT = Opportunity, GT = Growth, Interaction = CPECD∗FI, and PR = Poverty Reduction.

ΔR^2^ = % of total variance in the dependent variable accounted for by the model, R^2^ = % of total variance in the dependent variable accounted for by all the variables in the model together.

**Fig. 3:**
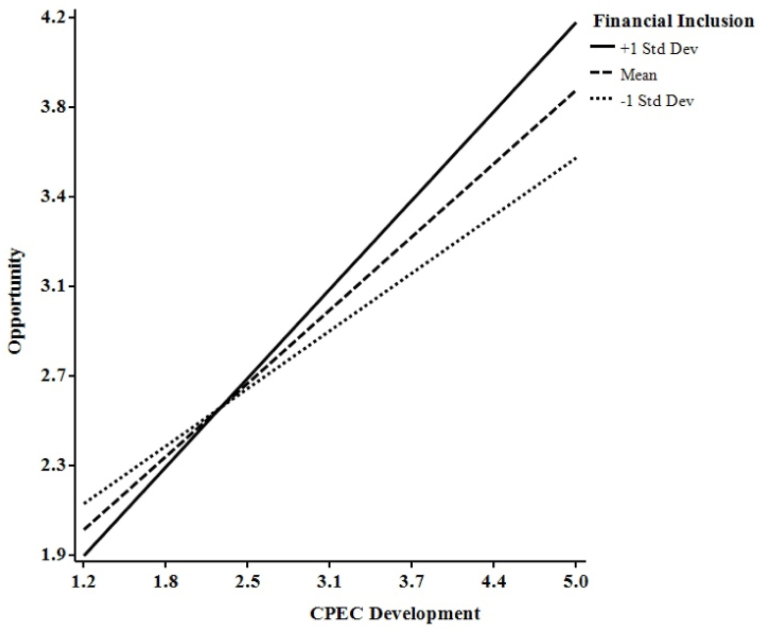
Interaction graph of opportunity.

**Fig. 4:**
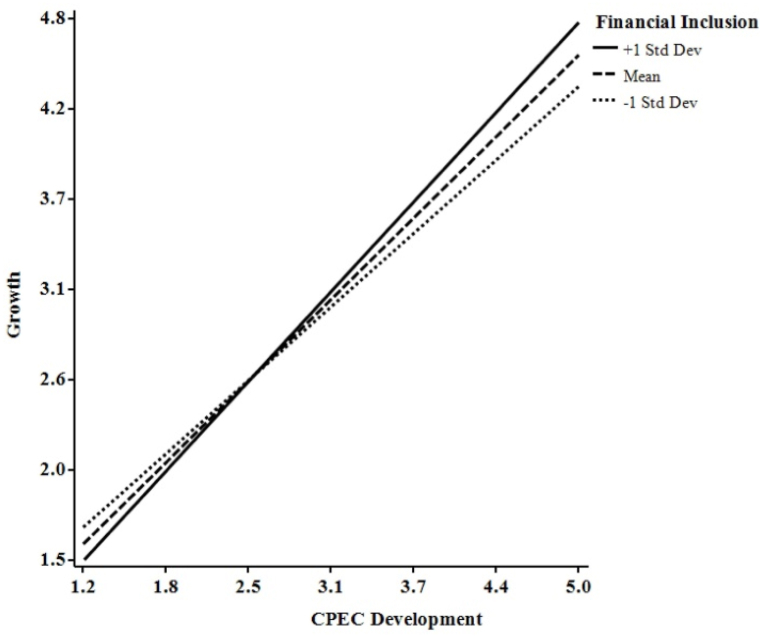
Interaction graph of growth.

Table 8 reveals that FI moderated the indirect influence of CPECD on PR via OT and GT. Higher FI levels predict higher OT and GT; lower FI levels predict lower OT and GT, as the conditional direct and indirect effects demonstrate. Table 9 also reveals that the conditional direct effects of FI on OT and GT are lower at lower FI values, higher at mean FI values, and highest at high FI values. Furthermore, the conditional indirect effects of FI on OT and GT were lower at lower FI values, higher at mean FI values, and highest at high FI values. Specifically, when FI rose, the impact of the interaction between CPECD and FI increased. Fig. 3, Fig. 4, as well as Table 9, show that CPECD had a greater impact on SMEs with a higher FI. Furthermore, when FI was at its highest, the effect on OT and GT was the strongest.

**Table 9:** Moderated conditional Analyses.

	Estimate	S. E	C.R	*p*-value	Lower	Upper
**Conditional Direct Effect (OT)**						
Lower value of FI	0.389	0.068	5.752	0.000	0.256	0.522
Mean value of FI	0.493	0.050	9.894	0.000	0.395	0.591
High value of FI	0.598	0.075	7.992	0.000	0.451	0.745
**Conditional Direct Effect (GT)**						
Lower value of FI	0.710	0.031	22.926	0.000	0.649	0.771
Mean value of FI	0.789	0.023	34.578	0.000	0.744	0.834
High value of FI	0.868	0.034	25.374	0.000	0.801	0.936
**Conditional Indirect Effect (OT)**						
Lower value of FI	0.092	0.021	–	–	0.053	0.136
Mean value of FI	0.117	0.023	–	–	0.074	0.164
High value of FI	0.141	0.030	–	–	0.085	0.205
**Conditional Indirect Effect (GT)**						
Lower value of FI	0.328	0.046	–	–	0.238	0.421
Mean value of FI	0.365	0.047	–	–	0.270	0.459
High value of FI	0.402	0.052	–	–	0.299	0.505

OT = Opportunity, GT = Growth, and FI = Financial Inclusion.

ΔR^2^ = % of total variance in the dependent variable accounted for by the model, R^2^ = % of total variance in the dependent variable accounted for by all the variables in the model together.

Following the suggestions of Cohen et al. [61], we plotted this interaction at conditional values of FI (one standard deviation above, below, and equal to the mean). The graphs show the interaction has a high, medium, and low influence. Fig. 3, Fig. 4 demonstrate how FI enhanced the connections between CPECD-OT and CPECD-GT and furthered the understanding of the moderation hypothesis. Fig. 3, Fig. 4 illustrate that low FI weakens the connections between CPECD-OT and CPECD-GT, while high FI strengthens them. Therefore, H5 was supported. It also suggests that firms with high FI were more likely to exploit economic opportunities compared to firms with low FI. To assess the significance of the interaction, the authors employ Jose's [62] approach, stating that the graph must not be parallel and have different slopes for the interaction to be significant. approach, which asserts that the graph must not be parallel and exhibit distinct slopes for the interaction to be statistically significant.

## 5 Results and discussion

### 5.1 Discussion

Based on social exchange and inclusive growth theories, this study examined the moderated mediational model. Here, the argument posited that the mediational effect of opportunity (OT) and growth (GT) in the relationship between CPEC development (CPECD) and poverty reduction (PR) was contingent on financial inclusion (FI). The researcher used empirical analysis to address the research question, *"Do CPEC development and financial inclusion drive trigger opportunity, growth, and poverty reduction?"*. In response to the research question, our study hypothesized that CPEC development significantly and positively impacts poverty reduction in Pakistan (H1). The empirical results confirm H1's acceptance. This finding demonstrates a link between increased CPECD and Pakistan's PR level. This empirical finding supports the cause-and-effect relationship between CPECD and PR. The study's findings are similar to those in the CPECD and PR literature. For example, Xie et al. [36] support these findings. They confirmed that the CPEC may create employment opportunities and alleviate poverty. Similarly, a recent empirical study by Kanwal et al. [17] shows the direct and indirect effects of perceived CPEC on quality of life through employment and education.

Furthermore, the researcher accepts H2a and H2b as supporting evidence for the indirect hypothesis (H2). Next, the researcher empirically tested the hypothesized mediating role of opportunity (H2). The researcher found that opportunity significantly mediates the relationship between CPECD and PR, indicating the acceptance of H2. This finding suggests that increasing opportunities will eventually improve Pakistan's PR level. This empirical evidence supports the hypothesized link's causation. Our findings are also comparable to those published by Saad et al. [7] and Kanwal et al. [63]. According to Saad et al. [7], CPEC has a favorable impact on quality of life, employment opportunities, poverty reduction, educational improvement, and environmental protection. Kanwal et al. [63] found that perceived economics, perceived education, perceived accessibility, and perceived employment all positively influence support for CPEC development via perceived income.

Moreover, the researcher accepts H3a and H3b, which support the indirect hypothesis H3. After that, the researcher tested the empirical findings for the growth mediator (H3). The findings revealed that growth significantly mediates the relationship between CPECD and PR. It signifies that H3 is accepted. This result suggests that increased growth contributes to improving Pakistan's PR level. This empirical evidence confirms and demonstrates the causality of the hypothesized link. Hassan et al. [38] endorsed this position and proposed that environmental, social, and economic factors mediate the positive association between concern about sustainable issues and sustainable development in the context of CPEC. Additionally, the most recent study by Rehman et al. [6] supports these findings, revealing that CPEC could enhance trade and transportation, thereby contributing to Pakistan's economic growth.

The researcher made a further effort to respond to the empirically analyzed hypothesis (H4) that financial inclusion moderates the mediated relationship of opportunity between CPEC development and poverty reduction. The findings demonstrated the significant moderating effect of FI in the association between CPECD, opportunity, and PR. It indicates that H4 is accepted. This result emphasizes that providing financial resources to SMEs in Pakistan accelerates the creation of various economic opportunities in the presence of higher CPECD and reduces poverty in Pakistan. The findings underline the importance of assessing the moderation effects of FI, as the conditional mediation impact of OT between CPECD and PR is higher when FI is higher. These findings match previous ones. For example, financial inclusion among entrepreneurs enhanced non-farm firm growth by 43 % in Ghana [64]. According to an International Monetary Fund (IMF) analysis, relaxing financial constraints on SME access to financing might enhance long-term cumulative growth by 5 % in specific MENAP nations, including the Middle East, North Africa, Afghanistan, Pakistan, and the Caucasus and Central Asia (CCA) countries [65].

Finally, the researcher tested the hypothesis (H5) that financial inclusion moderates the mediated growth relationship between CPEC development and poverty reduction. The findings demonstrated the significant moderating effect of FI in the association between CPECD, growth, and PR. It indicates that H5 is accepted. This finding stresses that a higher level of CPECD, together with much-needed finance for SMEs in Pakistan, promotes economic growth and helps to alleviate poverty in the country. In other words, when FI is high, the indirect positive effect of CPECD on PR is higher. Though not hypothesized, the moderation impact of FI was significant, but its effect size (GT ← Interaction = 0.063) was less for GT than for OT (OT ← Interaction = 0.083). This study validates the findings of Zada et al. [66], who claim that SMEs' growth and perceptions of contextual factors are significant predictors of their financing. According to their findings, access to finance paired with financial literacy enhances SMEs' growth more than a lack of access to finance. Donati [67] concurred with our findings, asserting that limited credit access hinders small enterprises' expansion and job creation. Extending financial inclusion allows previously excluded small businesses to obtain loans, increase production, and increase sales.

### 5.2 Conclusion

To recapitulate, finance and economics would not exist today if the link between megaprojects and PR was not critical. According to the literature on the impact of megaprojects like CPECD on PR, relevant authorities strongly emphasise encouraging and obtaining the required finance for communities. The CPECD-PR link is dynamic, multifaceted, and often fragile since CPECD effects must be transferred and translated across many levels of analysis over time. Contextual factors, such as distinct events, frequently alter the relationship. Data collection issues such as measuring relevant constructs, comprehending, moderating, and mediating mechanisms, and controlling for varied interpretations make investigations challenging. Recognizing all of these problems, we devised a comprehensive approach to comprehending the link between CPECD and PR. Our goal was not to create a comprehensive model that took into account all the important elements that could explain a relationship. For example, we eliminated previous material on the One Belt, One Road (OBOR) project in order to focus solely on items that improve our understanding of this link. We do not include reversal effects, such as how poverty reduction may benefit CPEC developments. We improve our understanding of the long-studied relationship between CPECD and PR by incorporating the most relevant literature from various theoretical perspectives (e.g., inclusive growth theory) to develop parallel mediation and moderated mediation models.

This research adds to the growing body of knowledge about CPECD and its reciprocal interactions with opportunity, growth, and PR. The study conducted an extensive analysis of the CPECD and financial inclusion (FI) across eight cities in three different regions of Pakistan: the north, central, and south. The research delved deeper than previous studies, aiming to provide a more comprehensive understanding of the subject matter. Opportunity, growth, and the PR have all been found to be intertwined with the CPECD to varied degrees. Two factors, opportunity and growth, were identified as important mediators of the study's findings. In other words, the study's findings can be attributed in large part to these elements. The research also found that financial inclusion has a substantial moderating role. This suggests that the link between the variables was significantly influenced by financial inclusion. Taking into account opportunity, growth, and financial inclusion appears to be crucial when analyzing the results of similar studies, as suggested by these findings. This research is groundbreaking since it is the first to examine the connections between the CPECD and financial inclusion. The study sheds light on how both of these factors can contribute to promoting inclusive growth and reducing poverty. Our research is expected to significantly impact how inclusive growth and poverty alleviation are conceptualized in the future. Additionally, it is likely to contribute to the body of knowledge on living standards.

### 5.3 Implications

Our findings have both theoretical and practical implications for the CPECD and PR literature.

I. Our analysis, which employs inclusive growth theory to analyze CPECD's effects on PR using FI as a moderator, supports and refines current understanding. Those who have concentrated on the CPEC-PR nexus have yet to consider FI's effects. By focusing on FI, our research contributes to the CPECD literature, particularly the CPECD-PR link.
II. Our study adds to the CPECD-PR literature by employing the mediators, namely opportunity and growth, as inclusive growth indicators. As a result, this study emphasizes the utility of inclusive growth theory in the CPECD literature and expands on putative mediators of the CPECD-PR link.
III. This study substantiates the moderating effect of FI on opportunity and growth in the link between CPECD and PR, suggesting new approaches to reduce poverty in real-world settings and highlighting the need for new strategies to improve access to financial services to ensure that individuals may benefit from economic expansion.
IV. The findings stress that through FI and optimizing resource allocation, inclusive growth principles might be better align with poverty reduction mechanism, addressing both social and economic exlusion.

### 5.4 Policy recommendations

Given its valuable findings, this study has made the following contributions: First, the significance of financial inclusion in reducing poverty in Pakistan provides evidence for future policies designed to promote the use of financial services. It has also identified a dire need to develop a formal financial system to increase financial inclusion and affect SMEs' growth. Second, the study also emphasizes FI's moderating effect. The research suggests that FI's intervention will expedite poverty alleviation through CPECD. Policymakers and stakeholders can use this information to boost FL, change current policies, and devise new policies. In turn, it may create trust in financial services and encourage more SMEs to use them. Finally, given that the CPECD has posed a challenge to the survival of Pakistan's SMEs, the research suggests that the government and financial institutions should develop effective interventions to assist small businesses struggling with financial resources to survive and compete. Consequently, it assists in achieving inclusive growth and poverty reduction.

### 5.5 Contribution

Based on the study's valuable findings, it is clear that it has made significant contributions. First, the findings contribute to a broader comprehension of the discourse on financial inclusion, particularly its importance for the CPEC-PR relationship. This study offers significant insights into how future policy efforts aimed at fostering a formal financial system and financial services might effectively enhance economic opportunities, promote economic development, and alleviate poverty. Second, the study brings attention to the impact of FI, a subject that previous research should have considered. Its involvement in CPECD will accelerate the poverty reduction effort by instilling individuals with greater financial independence. By doing so, individuals can invest in prospective opportunities such as research and development, emerging technologies, personnel training, education, and new business endeavours.

Third, this study offers insightful observations for policymakers and financial institutions regarding inclusive growth indicators, precisely opportunity and growth, in the context of the CPEC-PR link. It analyzes the indicators in mediational and moderated mediational models, which demonstrate their favorable trends in both models. Fourth, the research aims to assist the Pakistani government in overcoming challenges related to stagnant economic growth and impoverishment by providing insights on making megaprojects like CPEC more successful. Finally, other members of the One Belt, One Road initiative facing similar situations can benefit from the study's findings, which offer valuable insights into the role of CPECD, economic opportunities, and growth in PR.

### 5.6 Limitations and future research

Like any other research, it has some limitations. First, the directionality of linkages suggested in the hypothesized model is consistent with recent work on social exchange theory, inclusive growth theory, and CPECD and PR links. Cross-sectional data is not an optimal research design to determine causal order among hypothesized links. Thus, future studies may seek to replicate our approach using a longitudinal research methodology. This may yield more accurate causal inferences than this study. Second, this study used inclusive growth theory to explore OT and GT as the mediators between CPECD and PR. Using the same theoretical framework, future studies may examine a "trickle-down" effect of CPECD. Finally, we employed a novel moderator, FI; future research could test government measures and demographic variables.

## CRediT authorship contribution statement

**Salman Mahmood:** Writing – review & editing, Writing – original draft, Visualization, Validation, Supervision, Software, Resources, Project administration, Methodology, Investigation, Funding acquisition, Formal analysis, Data curation, Conceptualization. **Liangang Zhan:** Supervision. **Shoaib Aslam:** Writing – review & editing. **Navid Khan:** Writing – review & editing.

## Ethical considerations

The researcher adhered to the Helsinki Declaration on Research Ethics in this study. This study ensured maximum anonymity in addition to voluntary involvement.

## Statement of contribution

All listed authors made substantial contributions to the development and composition of this article.

## Data availability statement

The data supporting this research can be obtained from the corresponding author upon reasonable request.

## Declaration of competing interest

The authors declare that they have no known competing financial interests or personal relationships that could have appeared to influence the work reported in this paper.

## Figures and Tables

**Table: 3:** Exploratory Factor Analysis

Factor	Item loading	% Variance	% Cumulative Variance
CPEC Development	0.919- 0.933	30.665	30.665
Opportunity	0.867 - 0.916	18.885	49.550
Growth	0.722 - 0.846	16.902	66.453
Financial Inclusion	0.884 - 0.901	16.571	83.024
Poverty Reduction	0.763 - 0.882	2.925	85.949
Kaiser-Meyer-Olkin Measure of Sampling Adequacy is 0.886 Bartlett's Test of Sphericity is significant with 0.000, Approx. Chi-Square = 12828.434, and df = 276.			

**Extraction Method** = Principal Component Analysis and **Rotation Method** = Varimax with Kaiser Normalization.

## ACRONYMS

CPEC: China-Pakistan Economic Corridor
CPEC: China-Pakistan Economic Corridor Development
PR: Poverty Reduction
FI: financial inclusion
OT: Opportunity
GT: Growth
SDGs: Sustainable Development Goals
OBOR: One Belt, One Road
CCA: Caucasus and Central Asia
SMEs: Small and Medium Enterprises
SMEDA: Small and Medium Enterprises Development Authority
VIF: Variance Inflation Factor
CMV: Common Method Variance
S.D: Standard Deviation
EFA: Exploratory Factor Analysis
KMO: Kaiser-Meyer-Olkin
X2: Chi-Square
DF: Degrees of Freedom
SEM: Structural Equation Modeling
CFA: Confirmatory Factor Analysis
S.E: Standard Error
CR: Composite Reliability
C.R: Critical Ratio
AVE: Average Variance Extracted
RMSEA: Root Mean Square Error of Approximation
IFI: Incremental Fit Index
CFI: Comparative Fit Index
TLI: Tucker-Lewis Index
NFI: Normed Fit Index
DV: Dependent Variable
H: Hypothesis
AMOS: Analysis of Moment Structures
SPSS: Statistical Package for Social Sciences
SET: Social Exchange Theory
LB: Lower Bounds
UB: Upper Bounds

## Annexture 1

### Exploratory Factor Analysis (EFA)

Before analyzing the measurement and hypothesized model, we ran exploratory factor analysis (EFA) on five variables (CPEC development, opportunity, growth, financial inclusion, and poverty reduction). According to the EFA results, a five-factor answer explained 85.949 % of the overall variation. We deemed two opportunity (OT6 and OT7) and three growth (GT1, GT3, and GT7) items unacceptable in the current research setting because their factor scores were less than 0.40. They were removed from consideration [68]. Factor loadings ranged from 0.722 to 0.933, according to EFA. Bartlett's test = 12828.434, p < 0.001; Kaiser-Meyer-Olkin (KMO) = 0.886, over the needed 0.6 thresholds. Hence, it is an excellent match [68]. Following Table displays the EFA results.
