# Supplementary material for: Maximizing the impact of megaprojects: Urgent implications of financial inclusion drive for effective anti-poverty measures
Source: Heliyon. 2024 Oct 22;10(21):e39658. doi: 10.1016/j.heliyon.2024.e39658 (PMC11544048; doi:10.1016/j.heliyon.2024.e39658)
Supplement: Multimedia component 1 [file mmc1.pdf]

Appendix-A: Survey Questionnaire

This survey questionnaire is designed to assess the "Maximizing the Impact of Megaprojects: Urgent Implications of Financial Inclusion Drive for Effective Anti-Poverty Measures". This survey questionnaire has two sections (A and B). You are requested to notify the right information as per your knowledge. All information you provide will be kept strictly confidential and used only for academic and research purposes.

SECTION-A

You are requested to tick mark an appropriate option that best represents your feeling against each statement on Five-Point Likert Scale.

No.	Statement	(1) Strongly Disagree	(2)	(3)	(4)	(5) Strongly Agree
**CPECD**						
**1.**	CPECD1. CPEC should develop soon to improve the overall infrastructure and energy sectors.					
**2.**	CPECD2. CPEC development will attract more people and investment.					
**3.**	CPECD3. CPEC developments will play an important role in improving overall economy.					
**4.**	CPECD4. Development of CPEC is vital to the overall					

	development of Pakistan.					
**5.**	CPECD5. Supporting services development (travel agency, hotel, restaurants, entertainment, etc.).					

No.	Statement	(1) Strongly Disagree	(2)	(3)	(4)	(5) Strongly Agree
**Opportunity**						
**1.**	OT1. CPEC development provides individuals and SMEs the opportunities to start new business ventures.					
**2.**	OT2. CPEC development has increased employment opportunities.					
**3.**	OT3. CPEC development helps increase investment in Pakistan.					
**4.**	OT4. CPEC development has led to an improved business ecosystem.					
**5.**	OT5. CPEC provides the opportunities to integrate locals into society.					

No.	Statement	(1) Strongly Disagree	(2)	(3)	(4)	(5) Strongly Agree
**Growth**						
**1.**	GT2. CPEC development helps increase the demand for goods in the markets.					
**2.**	GT4. CPEC development helps to initiate and expand businesses.					
**3.**	GT5. CPEC development helps increase the overall productivity.					
**4.**	GT6. CPEC has led to the increase in the overall economy.					

No.	Statement	(1) Strongly Disagree	(2)	(3)	(4)	(5) Strongly Agree
**Financial Inclusion**						
**1.**	FI1. If credit is easily accessible then I can avail more CPEC opportunities.					
**2.**	FI2. I can participate in the growth process more efficiently if borrowings are					

	available from banks rather than money lenders.					
**3.**	FI3. To take loans I will prefer banks on money lenders, it will prevent us from the exploitation of money lenders.					
**4.**	FI4. Banking services such as consultations can help to take optimum advantages of loans under CPEC					
**5.**	FI5. Providing different banking services to the individuals under CPEC can give them economic and financial independence.					

No.	Statement	(1) Strongly Disagree	(2)	(3)	(4)	(5) Strongly Agree
**Poverty Reduction**						
**1.**	PR1. CPEC development would mitigate poverty across its lines.					
**2.**	PR2. CPEC development will increase the entrepreneurship, which					

	increases the income of local people.
**3.**	PR3. CPEC development will improve the personal income of the community.
**4.**	PR4. CPEC development will improve the economic conditions of the area which leads to a reduction in poverty.
**5.**	PR5. CPEC development will offer huge investment opportunities and improve the living standard of the people.

SECTION-B

Profile of Respondent:

Please put a tick mark to indicate your preference or write up your answer.

General Questions

1. Gender

   a. Male
   b. Female

2. Marital status

   a. Married

b. Unmarried

3. Age

a. 18 - 35 years
b. 35 - 55 years
c. Above 56 years

4. Education of SME owners

a. Below Matric
b. Matric
c. Intermediate
d. Graduate
e. Post Graduate
f. Diploma

Thank you very much for your time & cooperation.
